# Electric vehicle fire risk assessment framework using Fault Tree Analysis

**DOI:** 10.12688/openreseurope.16538.1

**Published:** 2023-10-18

**Authors:** Mohd Zahirasri Mohd Tohir, César Martín-Gómez

**Affiliations:** 1 Department of Construction, Building Services and Structures, Universidad de Navarra, Pamplona, Navarre, Spain; 2 Department of Chemical and Environmental Engineering, Universiti Putra Malaysia, Serdang, Selangor, 43400, Malaysia

**Keywords:** Electric vehicles, fire risk assessment, Fault Tree Analysis, state-of-the-art, safety, architecture, fire hazard

## Abstract

**Background:**

In the near future, the rapid adoption of electric vehicles is inevitable, driven by environmental concerns and climate change awareness. However, this progressive trend also brings forth safety concerns and hazards, notably regarding the risk of EV fires, which have garnered significant media attention. This necessitates the need to study for comprehensive fire risk assessment strategies aimed at preventing and mitigating such incidents.

**Methods:**

This study presents a framework for assessing fire risks in EVs using Fault Tree Analysis (FTA). By integrating disparate data sources into a unified dataset, the proposed methodology offers a holistic approach to understanding potential hazards. The study embarked on a comprehensive exploration of EV fire causes through qualitative FTA.

**Results:**

Through this approach, the work discerned five major causes: human factors, vehicle factors, management factors, external factors, and unknown factors. Using a meticulous weighted average approach, the annual EV fire frequency for each country was deduced, revealing an average annual EV fire rate of 2.44 × 10 ^-4^ fires per registered EV. This metric provides a significant benchmark, reflecting both the probability and inherent risk of such incidents. However, uncertainties in data quality and reporting discrepancies highlight the imperative of continued research.

**Conclusions:**

As EV adoption surges, this study underscores the importance of comprehensive, data-driven insights for proactive risk management, emphasizing the necessity for vigilant and adaptive strategies. The findings emphasize the pivotal role of this assessment in shaping response strategies, particularly for first responders dealing with EV fires. In essence, this research not only elevates the understanding of EV fire risks but also offer a foundation for future safety measures and policies in the domain.

## Abbreviations

Battery Electric Vehicles (BEVs)

Battery Energy Storage System (BESS)

Danish Institute of Fire and Security Technology - Dansk Brand- og sikringsteknisk Institut (DBI)

Electric vehicle (EV)

Fault Tree Analysis (FTA)

Fuel Cell Electric Vehicles (FCEVs)

Greenhouse Gases (GHG)

Hybrid Electric Vehicles (HEVs)

Internal Combustion Engine Vehicles (ICEVs)

Online Data and Reporting System (ODIN)

Plug-in Hybrid Electric Vehicles (PHEVs)

Solid-electrolyte Interphase (SEI)

Swedish Civil Contingencies Agency - Myndigheten för samhällsskydd och beredskap (MSB)

## Introduction

Environmental concerns on land transport emissions and awareness towards climate change have stemmed governing bodies around the world to come out with transport regulations which supports the deployment of electric vehicle (EV). In general, the European Union and its member states have committed to a binding target to the reduction of greenhouse gases (GHG) of at least 40% by the year 2030 as compared to 1990 ^1^. To achieve this goal, recently, the European Union have announced several policies towards emission standards for land vehicles starting from the year 2020. The standards include emission target for new cars which is at 95 gCO2/km, target of 90% reduction in transport greenhouse gases (GHG) emissions by 2040, and projection of 13 million zero- and low- emission vehicles by 2025 ^2^. On top of this, several countries in Europe have announced its own policies towards promoting electric vehicle deployment ^3– 7^. As a result of the supportive policies along with rapid technological advances for EVs, the global EV stock has increased by an annual average of 60% between the year 2014 and 2019 ^8^. In the year 2019, global EV sales reached 2.1 million which is a 6% increase from the previous year ^8^. In Europe, the market share of EVs has increased to 10% in the year 2020 as compared to only 3.2% in the year 2019 ^8^. This trend is expected to increase in the near future.

However, this positive trend comes with safety risks and hazards associated with EVs. One significant safety risk is the burning of EVs, which has garnered considerable interest, particularly from the media. Over the past few years, numerous cases of EV fires have been reported globally, drawing significant attention. Some notable incidents that have recently occurred include the one in March 2023. A car transporter ship, carrying almost 4,000 vehicles, including electric ones from Volkswagen, Porsche, and Audi, sank after an onboard fire, which started from the cars' lithium-ion batteries ^9^. In February 2023, a parked Tesla Model S in San Francisco ignited due to its battery, but the fire was controlled by its built-in suppression system ^10^. In the same year, a Ford F-150 Lightning caught fire in Michigan; Ford stated it wasn't battery-related and was investigating the cause ^11^. From these recent incidents, it shows that the concerns on the burning risks of electric vehicles are valid.

Though, according to a study by The Danish Institute of Fire and Security Technology or Dansk Brand- og sikringsteknisk Institut(DBI) ^12^, electric and hybrid vehicles have a lower probability of catching fire compared to those with internal combustion engines. Nonetheless, this study by DBI only analysed the statistics from Norway without providing any details about the analysis. Hynynen *et al.* attempted to collect electric and hybrid vehicle incidents from several countries, namely, Norway, Sweden, Denmark, the United States, and China ^13^. The authors were aware that due to the comparatively lower number of EVs to internal combustion engine vehicles (ICEVs), the available statistical data for EVs is still limited. In their work, they concluded that the collected data indicates that EVs are 8–10 times less likely to catch fire than ICEVs, though this may change as more EVs age. In the review of methodological approaches, it is proposed that electric vehicles, especially when parked or stationary, can be conceptualized as Battery Energy Storage Systems (BESS). This perspective aligns with the American NFPA 855/2020 standard, which provides an assessment of related risks ^14^. However, the standard suggests a need for further refinement and elaboration. Strategies encompassing active, passive, and maintenance measures, as detailed in the work by Blanco-Muruzábal *et al.*, offer potential avenues for enhancing the evaluation framework ^15^. More detailed fire statistics, including root causes and battery involvement, are recommended for better analysis as current data lacks specifics on the energy carrier.

Building on this need for comprehensive data, pertaining to the possible causes of ignition, up until now, there are concerns on the fire safety of EVs due to thermal safety issues in battery systems used ^16^. Batteries in EVs usually consists of thousands of cells configured either in series or parallel to satisfy the energy and power demand. The increased number of cells corresponds to the increasing storage capacity of the energy, while at the same time intensifies the detrimental effect should any safety issue occurs ^16^. Previous studies have shown that batteries used in EVs are susceptible to thermal runway which could lead to whole vehicle to burn ^17^. Some notable fire incidents involving EVs were associated to batteries ^17, 18^. Even though the battery is found to be the major cause of EV fire incidents, there are other factors that can possibly cause an EV fire incident such as the spontaneous ignition due to arson or sustained abuse, fire during the charging process, self-ignition while in driving and fire after traffic collisions ^18^. Consequently, given the complex and diverse nature of EV fires causes, coupled with rapidly changing technology, achieving a reliable assessment of fire risk through traditional analytical methods proves to be a significant challenge.

In light of this, the Fault Tree Analysis (FTA) is a critical tool in proactively preventing failures and can also be used to assess and improve system risk levels at any stage. It is a single event-oriented method that visually represents the interplay and dependencies between hazardous events and their root causes, including human error, component failure, and varying environmental and operational conditions ^19, 20^. FTA have been used for safety engineering related applications in the past particularly in identifying potential failure points within a system and assessing their possible impact. By illustrating the logical interrelations of failures, it enables engineers to proactively design safeguards and preventive measures, improving overall system safety. Moreover, it's been crucial in sectors like nuclear power, aerospace, and chemical industries, where understanding the cascading effects of single-point failures is vital for catastrophic risk mitigation. Recent FTA applications for safety risks include Mohd Nizam Ong *et al.*'s study on fire risks in rooftop PV systems, pinpointing arcing from human error and poor quality control as the chief ignition sources ^21^. While another work by Zermane *et al.* used FTA to evaluate risks of fatal accidents from falling, integrating statistical data analysis for proactive prevention ^22^. Their approach also incorporated statistical analysis of collected data as part of a dual risk assessment method. To conclude, FTA provides a practical technique to proactively identify and assess potential failure points within complex systems, offering insights that can inform safety measures and improve overall risk management.

Using the general definition of risk, the risk of EV fire is the product of the ignition probability multiplied by the consequence in case of ignition. There's no question that lessening the probability of ignition will decrease risk, yet focusing more on lessening the consequence should ignition occur could have an even more substantial effect on the overall risk. The proposed risk framework is aimed at unravelling the root causes of EV fires, leveraging all accessible datasets. This could help determine whether reducing the ignition probability or mitigating the consequences would result in the most significant reduction in fire risk of EVs.

This study focuses on developing a comprehensive risk assessment framework capable of predicting the quantitative frequency of EV fires and to delve into both the qualitative and quantitative aspects of EV fire causes using FTA. By laying out the fire-related failure patterns in EVs, the output of the work aims to equip industry players like regulators, designers, manufacturers, installers, and users with an understanding of root causes, thereby allowing them to introduce effective preventive measures to curtail human and property losses. This study, therefore, acts as the foundation for a more in-depth exploration of EV fire risks in times to come.

## Methodology

This work presents a comprehensive framework for risk probability assessment of EV fires. Two important aspects of the methodology are the development of the risk assessment framework for EV fire and the data acquisition process.

### Development of risk assessment framework for EV fire

For the purpose of this study, a consistent definition of EV has been adopted. An EV is a vehicle that operates using one or more electric motors for propulsion, deriving its power from electricity stored in batteries or another energy storage device. Unlike traditional internal combustion engines vehicles that run on gasoline or diesel, EVs utilize electricity, which can be sourced from renewable energy, nuclear power, fossil fuels, or any combination thereof. This electricity is typically generated off-site and is transferred to the vehicle through a charging station or wall outlet, then stored in the vehicle's onboard batteries. There are different types of electric vehicles, including: Battery Electric Vehicles (BEVs): These are purely electric vehicles with no gasoline engine. They run entirely on electricity and are powered by one or more electric motors, which get energy from onboard batteries. Once the batteries are depleted, they must be recharged. Plug-in Hybrid Electric Vehicles (PHEVs): These vehicles have both an electric motor and a traditional gasoline or diesel engine. They can operate on electricity for shorter ranges and switch to their internal combustion engine or use both when the battery is low or when additional power is needed. Hybrid Electric Vehicles (HEVs): Similar to PHEVs, HEVs have both an electric motor and an internal combustion engine, but the difference is that HEVs cannot be plugged in to charge their batteries. Instead, the batteries are charged through regenerative braking and by the internal combustion engine. Fuel Cell Electric Vehicles (FCEVs): These vehicles use hydrogen gas to power an onboard fuel cell, which produces electricity to run the motor. They emit only water vapor and heat, making them a zero-emission vehicle, similar to BEVs. In this work, the term EV is limited to road passenger vehicles as other types of vehicles pose different risks.

The fault tree analysis was utilized as the risk assessment framework to identify possible root causes of fires related to EVs. This process includes identifying specific events and their associated faults, as well as establishing the relationships between these events to form various branches in the tree. The qualitative fault tree analysis aids in determining a range of potential causes for the top event by identifying major, intermediate, and basic events. All these events were linked to the top event via logical gates. All the possible hazards and causes of fires were identified and supported by literature. For the quantitative fault tree analysis, the failure rate data was required, correlating the number of fires with the cumulative EVs registered for a given year. In addition, the contribution of a specific EV fire causes was gauged by the frequency of fires it instigated. The main outcomes from both the qualitative and quantitative fault tree analysis were extensively discussed in this step.

### Data acquisition

**Figure 1.**
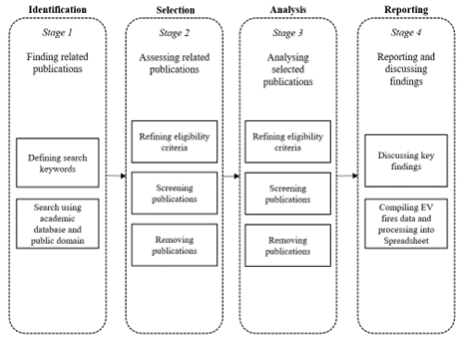
Stages of systematic literature search in this study.

This study utilizes a systematic literature search to gather data, drawing inspiration from the methodology proposed by Ramali *et al.* ^23^. By designing pertinent search queries at the outset, the authors ensured a focused direction for our research, enabling the authors to identify and analyze crucial data on EV fires statistics from select academic databases (Scopus and Google Scholar) and public domains (Google). The decision to include public domains stems from the fact that some academic databases do not archive reports authored by various global agencies and institutes. The search focuses on sourcing related publications such as research articles, technical reports, incident reports on EV fire investigations, and open-source data from various countries. Figure 1 illustrates the review process undertaken in the research to collate pertinent documents and extract salient findings on EV fires. During the identification phase, seven keywords, namely: "EV", "fire", "statistics", "data", "country", "report", and "incident" were combined to formulate the query strings. Among these, "EV" and “fire” were identified as a requisite keyword. These central keywords, “EV” and “fire” were systematically paired with the other five terms to target statistics related to EV fires. To optimize accuracy during the search, the authors utilized the advanced search capabilities of the engine, integrating Boolean operators like "AND" to filter through the designated academic databases. A document was deemed relevant when the search string or associated keyword appeared within its content. Table 1 provides a breakdown of the number of documents retrieved from the literature search.

**Table 1.** Search outcome from the literature search.

Search strings	No. of documents found in Scopus, Google Scholar and Google
“ev AND fire AND statistics”	16
“ev AND fire AND data”	61
“ev AND fire AND incident AND report”	10
“ev AND fire AND country AND incident AND report “	0
“ev AND fire AND country AND report”	1

The initial search yielded a total of 88 documents sourced from Scopus, Google Scholar, and Google (see data availability statement). During the selection phase, documents were meticulously screened against specific exclusion criteria. This included the elimination of duplicates, publications dated before 2010, conference reviews, patents, cover pages, and inaccessible documents. Moreover, only those documents with a clearly identified data source were retained. Any document meeting these exclusion criteria was omitted. A conclusion from the literature search has found that most of the research papers discussing EV fire data eventually comes from public domain reports. An example of this was demonstrated by a paper by Hassan *et al.* uses data from public domain reports to analyse EV fires in Australia ^24^. As a result of the literature search, reliable data on EV fires was predominantly found in public domains. Consequently, the authors decided to focus on data sourced from these public platforms, namely, Denmark ^25^, Republic of Korea ^26^, The Netherlands ^27^, Norway ^28^, Sweden ^29^ and Finland ^30^.

The next step is to process this data. Due to national variations, the data were not uniform, necessitating the reorganization and harmonization of the data. Consolidating incident data from different sources is critical for accurate and comprehensive risk assessment. Given the varied ways in which such data may be reported across diverse databases, it's essential to normalize it for improved processing and understanding. When dealing with time-series data specifically, which tracks incidents over a period of time, consolidation becomes even more vital. The different sources could contain varying levels of detail, be subjected to different reporting standards, or have diverse methods of categorization. By integrating this disparate information into one dataset, the work can ensure consistency in how the data is analysed and interpreted. In summary especially when data is scarce, the process of consolidating electric vehicle incident data from various sources enhances our ability to understand and interpret the data, facilitates effective risk assessment, and ultimately guides the creation of strategies to mitigate potential hazards. Despite the scarcity of the data, the analysis is considered crucial as it provides a global risk assessment related to EVs fires.

In a further analysis, datasets indicating the percentage of fires caused by specific EV fire causes were only accessible from three countries: Denmark ^25^, The Netherlands ^27^ and Sweden ^29^. As these datasets are not standardized amongst each other, the origins of failure leading to an EV fire were categorized based on the major events identified in the fault tree analysis. The average percentage of components causing fires in the EVs was then normalized based on the frequency of incidents related to specific components, obtaining the number of fires per million vehicles per year by the EV fire cause. Even though the current data stems mostly from Europe, until more data is available, it is plausible to consider the results to be similar in other continents. However, it's important to note that no continent can be considered as a homogeneous region, given the differing stages of technological development from one country or region to another.

The data from Denmark was obtained from a report that focuses on fire incidents in EVs and hybrid vehicles for the year 2018 – 2021 ^25^. The data for this analysis was sourced from the rescue service's Online Data and Reporting System (ODIN), spanning the period from 1 January 2018 to 30 September 2021. The causes of incidents were explicitly identified and mentioned in the report. Next, the data from the Netherlands was obtained from a report by Nederlands Instituut Publieke Veiligheid ^27^. Out of all the incidents recorded, only 36 of the incidents have been reported to include its possible causes, hence, these numbers were used for the analysis in this work. Finally, the data from Sweden was taken from a report by Swedish Civil Contingencies Agency or Myndigheten för samhällsskydd och beredskap (MSB). This report publishes a compilation of fires in electric vehicles and electric means of transport in from the year 2018 until 2022. All the possible causes of incidents are published in the report.

### Results and discussions

#### Fault Tree Analysis

**Figure 2.**
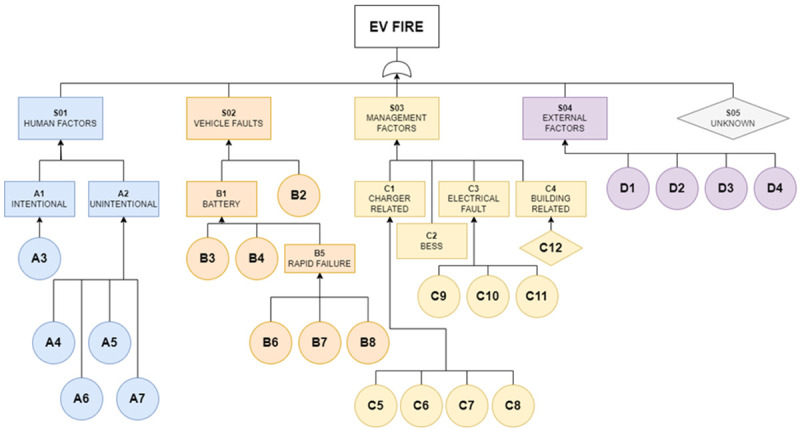
Complete standard qualitative Fault Tree Analysis Diagram for EV fire.

***Qualitative fault tree analysis.*** In the fault tree analysis, the top event under consideration is EV fires, which represent the ultimate outcome being assessed. This top event branches into four major identifiable causes - human factors, vehicle faults, management factors, and external factors. A fifth major event, which encapsulates unknown causes of ignition, is also included but remains undeveloped in the tree. These five major causes, constituting both developed and undeveloped events, further diverge into seven intermediate and 22 basic events. Since any of these major events could independently trigger the top event, they are interconnected with 'OR' gates. The identifier (ID) used in this qualitative fault tree analysis diagram are detailed in Table 2 and Figure 2 shows the standard qualitative fault tree analysis diagram.

**Table 2.** Fault tree analysis events and codes linked with the fault tree analysis diagram in Figure 1.

ID	Event	ID	Event	ID	Events
S01	Human factors	B3	Defect	C9	Short circuit
S02	Vehicle faults	B4	Degradation	C10	Trip
S03	Management factors	B5	Rapid failure	C11	Other
S04	External factors	B6	Mechanical abuse	C12	Other
S05	Unknown	B7	Thermal abuse	D1	Animals
A1	Intentional	B8	Electrical abuse	D2	Firebrands
A2	Unintentional	C1	Charger related	D3	External building fire
A3	Arson	C2	BESS	D4	Natural phenomenon
A4	Crash	C3	Electrical fault		
A5	Hot work	C4	Building related		
A6	Negligence	C5	Work near live electrical equipment		
A7	Smoking	C6	Operating above safe limits		
B1	Battery	C7	Failure of equipment		
B2	Other parts	C8	Ignition of flammable fuels at charger		

**S01 Human factors**

The first major event, human factors are referred to as ergonomics, encompass all the elements that influence the interaction between humans and the systems they use ^31^. Human factors leading to EV fires encompass actions, behaviours, or conditions attributable to human interaction that can increase the risk of fire in these vehicles. In this work, the human factors are divided into two intermediate events which are intentional and unintentional. In the intentional category, arson is identified as a basic cause that can lead to a fire. While there are no reports of arson on EV, there is always a possibility that this will happen in the future.

For unintentional fires, the causes are expanded into four basic causes, crash, hot work, negligence, and smoking. A4 refers to crash, which is an unwanted event that is identified to lead to an EV fire. Like other types of vehicles, EV are also prone to crashes due to various human factors including driver error, distracted driving, reckless behaviour, impaired driving, and failure to follow traffic rules, among others ^32^. A5 refers to hot work which encompasses tasks that generate heat, flames, or sparks. Within the automotive industry, this includes procedures like welding on body components, making electrical solder connections, operations involving cutting, or any repair tasks that emanate heat or spark. A6 refers to negligence, in the context of EVs, this pertains to any actions or inactions that amplify the potential for a fire outbreak. Such lapses might encompass inappropriate handling or usage, inadequate maintenance of the EV, or overlooking the safety directives stipulated by the vehicle maker. A7 refers to smoking: smoking within or near to an EV could pose an increased fire risk, particularly when careless practices are observed. Cigarettes, cigars, or other smoking materials can create a fire hazard when they encounter flammable materials ^33^. Inside a vehicle, these materials can include fabric upholstery, seat covers, floor mats, or other flammable interior components.

To effectively prevent and avoid EV fires, it's crucial to understand and address these basic causes. In the case of unintentional causes, strategies might involve improving vehicle design and manufacturing standards, promoting safe charging practices, and implementing robust vehicle monitoring and safety systems. For intentional causes, solutions may focus on raising awareness about the dangers of reckless behaviour, strengthening legal deterrents, and improving surveillance and security measures for EVs.

In both cases, consolidating and analyzing incident data from various sources can provide valuable insights into the specific factors contributing to EV fires, helping to identify potential areas of focus for preventive measures and to continuously improve the safety and reliability of EVs.

**S02 Vehicle factors**

The second major event, termed ‘vehicle factors’ is defined as the issues relating to inherent characteristics, conditions, or faults within the vehicle itself. Vehicle factors contributing to EV fires can largely be divided into two intermediate causes: issues related to the battery and issues related to other vehicle components.

The battery issues can be extended to two basic causes i.e. defect and degradation and one intermediate cause i.e. rapid failure. B3 refers to defect: These can stem from the manufacturing process or design of the battery ^18^. Manufacturing defects might include contaminants in the battery cell, poor quality control, improper alignment of components, or insulation failure ^34^. Any of these could potentially lead to internal shorts or other malfunctions, causing the battery to overheat and potentially ignite. Design defects might include inadequate safety features, improper thermal management, or insufficient protection against overcharging.

B4 refers to degradation: All types of batteries experience degradation over time, characterized by a gradual loss of capacity and increase in internal resistance ^35^. Battery degradation stems from physical and chemical changes within the cell, primarily observed as capacity fade (a reduction in the usable capacity) and power fade (a reduction in the deliverable power). Key degradation modes include loss of active material, reduction in available lithium for transport between electrodes, and changes in impedance or resistance. Common mechanisms causing degradation encompass solid-electrolyte interphase (SEI) formation, lithium plating, particle fracture, and the interaction between various degradation processes. As batteries degrade, the structural and chemical alterations can compromise the cell's integrity, potentially leading to internal short circuits, overheating, or thermal runaway, all of which elevate the risk of fires.

B5 refers to rapid failure: This intermediate cause generally refers to sudden catastrophic battery failure, often involving thermal runaway ^36^. Rapid failure can be triggered by severe mechanical, thermal, and electrical abuse in which can be further derived to be the potential basic causes of EV fire ^37^. The first basic cause is B6 refers to mechanical abuse: This refers to any form of physical damage to the battery, which can be caused by accidents, mishandling, or poor maintenance ^38^. Mechanical abuse could result in deformation of the battery components or even rupture of the casing. For example, a severe car crash could physically damage the battery pack, potentially causing an internal short circuit or a breach of the battery's protective casing. Both could lead to rapid failure, such as thermal runaway, which can in turn cause a fire. B7 refers to thermal abuse which is exposing the battery to extreme temperatures, either too high or too low ^36^. Overheating can be particularly dangerous as it can cause the battery's electrolyte to break down, leading to a build-up of gas and potentially causing the battery to explode ^36^. For example, if a battery overheats, the breakdown of its electrolyte can produce excess gas, potentially leading to an explosion and subsequent fire. A well-designed thermal management system is crucial in an electric vehicle to avoid such issues and to keep the battery operating within its ideal temperature range ^39^. B8 refers to electrical abuse: This typically refers to inappropriate charging or discharging conditions, such as overcharging, undercharging, or rapid charging/discharging beyond the battery's design limits ^36, 40^. Overcharging can cause lithium-ion batteries to heat up and could potentially lead to thermal runaway ^40^. For instance, overcharging a lithium-ion battery can induce excessive heat, pushing it towards thermal runaway, which can ultimately spark a fire, highlighting the perils of electrical misuse ^41^. Rapid charging or discharging can strain the battery and accelerate degradation, increasing the risk of failure.

By understanding these basic causes, strategies for prevention and mitigation can be formulated and implemented. This includes designing robust safety systems, improving manufacturing practices, implementing rigorous quality checks, and raising awareness among users about proper maintenance and operation. Also, data collection and analysis from real-world incidents can help in identifying patterns and refining preventive measures over time.

**S03 Management factors**

The third major event are management factors that relate to the operational processes, policies, and maintenance practices related to the vehicle. Management factors can significantly contribute to EV fires, and these can be broken down into four main intermediate causes: charger-related incidents, battery energy storage system (BESS), electrical faults, and building-related issues.

C1 refers to charger related incidents: Chargers for electric vehicles need to handle high levels of electrical power, making them potential fire hazards. Basic causes that can lead to charger-related incidents include working near live electrical equipment (C5), operating chargers above safe limits (C6), equipment failures (C7), and ignition of flammable fuels at the charger (C8). Enhancing safety protocols around charger use, enforcing safe operational limits, regular maintenance, and ensuring the charging area is free from flammable materials can minimize these risks. C2 refers to battery energy storage system (BESS): The BESS of an electric vehicle is intricate and requires effective management to maintain safety. Poor design, substandard manufacturing, and misuse are potential basic causes of failure. By improving design and manufacturing processes, conducting regular maintenance, and educating users, the risk of BESS-related fires can be reduced.

C3 refers to electrical faults: Electrical faults are a primary concern in EV fire incidents. Basic causes of such faults can include short circuits and tripping. These issues could originate from manufacturing defects, wear and tear, lack of maintenance, or improper installation. Regular inspections and maintenance, effective design and manufacturing practices, and proper installation can significantly reduce the risk of such faults leading to fires. C4 refers to building-related issues: Issues related to building structures, including insufficient fire safety precautions or inherent design flaws, can increase the risk of EV fires during charging sessions inside these buildings.

By identifying these basic causes, efforts can be focused on improving safety protocols, enhancing vehicle design, and educating users to effectively prevent and avoid fires related to electric vehicles.

**S04 External factors**

The fourth major event is external factors which are typically beyond the control of vehicle manufacturers, owners, or operators. External factors also pose a significant risk of leading to EV fires. These can generally be categorized into four basic causes: animal interference, firebrands, external building fires, and natural phenomena.

D1 refers to animal interference: Certain types of animals, such as birds or rodents, can pose an external fire risk to electric vehicles. These animals may carry flammable materials, such as dried grass or twigs, for their nests, which could be left near or inside the vehicle. Additionally, some larger animals may knock over external heating sources or ignite flammable materials through other means. These situations could potentially cause a fire that might spread to the vehicle. To prevent such scenarios, it is recommended to park vehicles in areas that are not easily accessible to wildlife, and to regularly inspect and remove any accumulated materials near the vehicle.

D2 Firebrands: Firebrands are burning pieces of airborne wood or vegetation that can land on or near an EV, posing a fire risk ^42^. The threat is especially significant in areas prone to wildfires. Vehicles should be parked away from vegetation, and in covered areas during wildfire events, to minimize exposure to firebrands. D3 refers to external building fires: If an EV is parked inside or near a building that catches fire, it could also be set alight. Keeping the vehicle in well-maintained, fire-resistant structures, and ensuring proper fire safety measures in the building (like smoke detectors and fire extinguishers), can mitigate this risk. D4 refers to natural phenomena: Certain natural events like lightning strikes or extreme weather conditions can cause fires in EVs ^43^. While such incidents are largely unpredictable, keeping vehicles sheltered during extreme weather and installing lightning protection systems in areas prone to such events can offer some level of protection.

Understanding these external factors and implementing preventive measures can significantly reduce the risk of EV fires. Regular inspection, careful storage, and increased awareness of environmental threats are all key elements of fire prevention for electric vehicles.

***Quantitative fault tree analysis.*** In this section, the collected incident data are analysed and discussed as to provide insights of the current trends of EV fires from the involved countries. The primary intent of this analysis is to determine the failure rates triggering EV fires and identify the predominant causative factors. Quantitative fire risk in this investigation is defined by the annual count of national EV-related fires, balanced against the sum of registered EVs within the country. The choice of this approach is due to the expectation of increasing number of fire cases with the number of corresponding registered EVs in a country. This approach is supported by the qualitative fault tree analysis from the previous section where it was understood that the presence of more EVs will potentially cause more EV fires. As more countries factor EVs into their post-fire assessments as potential ignition sources, and as the existing data-contributing nations supply more information, the analysis will naturally evolve and enhance its accuracy. Therefore, this following quantitative fault tree analysis represents the most comprehensive and reliable quantitative examination achievable with the accessible data as of 2022.

**Figure 3.**
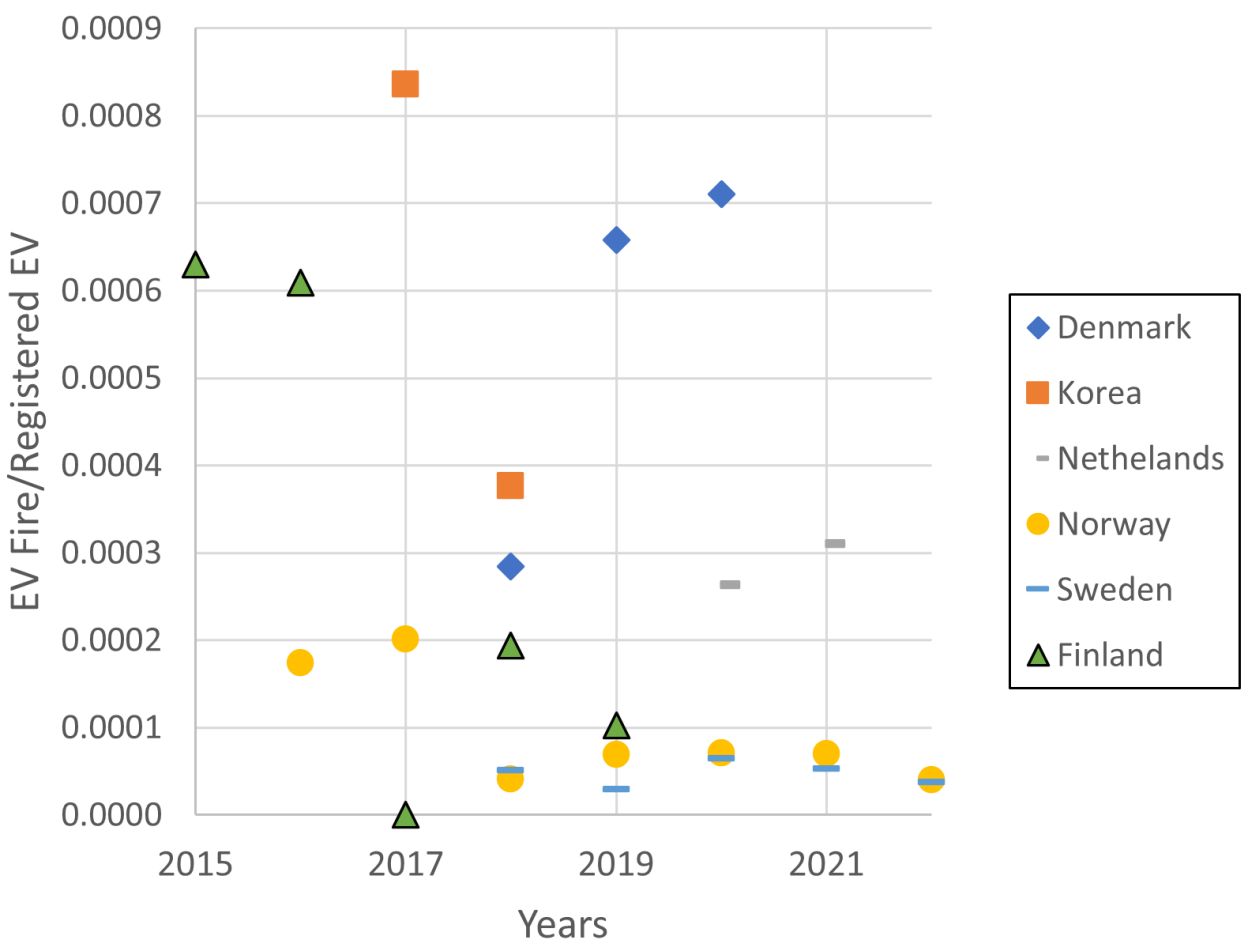
The annual frequency of EV-fires for the six countries over recent years.

Datasets from six countries were collated and Table 3 summarizes the number of fires, the cumulative number of registered EVs in its corresponding countries, the number of EV fires per registered EVs, the weighted average of EV fires per registered EV per year and the overall weighted average for all datasets. The annual frequency of EV-related fires, based on data presented in Table 2, is graphically depicted in Figure 3, clearly illustrating the stark disparity among the four countries examined. There are no obvious trends over year of incidents based on EV fires per registered EVs in all six countries. Apart from a solitary instance, neither Netherlands, Sweden and Norway exceed a frequency of 3.00 × 10 ^-4^ fires per MW per year, in contrast to Korea, Denmark and Finland where the numbers are generally inconsistent. This notable difference can be primarily attributed to the way the data is collected. A work by a consortium led by Efectis in a European Union project outlines the problems with various countries defining and interpreting information prior entering it into incident report system ^44^. This has led to potential bias in the data collected from different countries. From the limited data, there are no clear trends of incidents as the year progresses.

**Table 3.** Input data and calculation of the overall number of EV fires per registered EV per year.

Country	Year	Number of Fires	Cumulative of registered EV	EV Fires/Registered EV	Weighted Average EV Fires/Reg EV/year	Overall weighted average (EV fires/Reg. EV/year)
**Denmark**	2018	3	10541	2.85E-04	6.52E-04	2.44E-04
	2019	10	15205	6.58E-04		
	2020	18	25345	7.10E-04		
**Korea**	2017	21	25108	8.36E-04	6.07E-04	
	2018	21	55756	3.77E-04		
**Netherlands**	2020	71	270303	2.63E-04	2.92E-04	
	2021	118	381335	3.09E-04		
**Norway**	2016	17	97532	1.74E-04	9.97E-05	
	2017	28	138983	2.01E-04		
	2018	8	195351	4.10E-05		
	2019	18	260692	6.90E-05		
	2020	24	340002	7.06E-05		
	2021	32	460734	6.95E-05		
	2022	24	599169	4.01E-05		
**Sweden**	2018	8	156331	5.12E-05	4.96E-05	
	2019	6	207904	2.89E-05		
	2020	20	308485	6.48E-05		
	2021	24	452413	5.30E-05		
	2022	23	610716	3.77E-05		
**Finland**	2015	1	1587	6.30E-04	3.04E-04	
	2016	2	3285	6.09E-04		
	2017	0	7168	0.00E+00		
	2018	3	15499	1.94E-04		
	2019	3	29364	1.02E-04		

In this analysis, a weighted average is used to calculate the annual EV fire frequency of each country. Eventually, considering the data from the six countries, an average annual EV fire frequency of 2.44 × 10 ^-4^ fires per registered EV is produced from the analysis by using a weighted average, factoring in the annual fire count. In terms of per million units, the EV fire frequency is determined to be 244 fires per million registered EVs. This is significantly higher compared to the average value of 5.29 EV fires per million registered EVs reported by Hassan *et al.* ^24^. The substantial discrepancy between these values can be attributed to the method employed to calculate the fire frequency. In Hassan *et al.'*s study, fire frequency was computed by summing all EV fire incidents for a specific year, irrespective of location, and then dividing by the global number of registered EVs. In contrast, this study utilizes a weighted average in place of the traditional arithmetic average. This approach ensures that each incident is equally represented, preventing the skewing of data from countries with thorough and consistent reporting systems. Nevertheless, the frequency is an important finding as it provides data-backed information of possible estimation of EV fires based on number of EV registered. Given the uncertainties surrounding the data quality and the potential discrepancies in fire incident reports, this challenges the necessity for further analyses.

The International Energy Agency forecasts that by 2025 and 2030, the number of EVs on European roads will approximate 21.3 million and 56 million, respectively. Given the average annual frequency of 2.44 × 10 ^-4^ fires per registered EV, we can project around 5,194 EV fires in Europe for 2025 and about 13,655 for 2030. These figures are cause for concern, even if they might lean towards overestimation. While advancements in technology are expected to enhance fire safety measures, potentially reducing the real number of incidents, one thing is clear: as the number of EVs on the roads grows, so will the occurrence of EV fire incidents.

In this analysis aimed at ascertaining the frequency of incidents tied to specific causes, only intermediate causes were taken into account. This decision was influenced by the limitations inherent in the data sources, which originated from Denmark, The Netherlands, and Sweden. It's imperative to understand that each of these countries adopts its own unique method for collecting and registering data. Given these disparities in data collection methodologies, ensuring a consistent cross-country analysis became challenging. To overcome this and provide a consistent frame of reference, the authors classified the various causes into intermediate causes. This approach even though with limitations, proved instrumental in identifying which causes are pivotal to focus on in addressing the issues of EV fires moving forward.

Further analysis into the causes of EV fires, Table 4 shows the collated data of identified major causes for the three countries; Denmark, Netherlands, and Sweden; and its normalised average percentage of initiating causes of an EV fire. Given the limited data available, this analysis offers a preliminary insight into the initiating causes of EV fires. The table also presents the frequency of major causes that can lead to EV fire based on the available data. Recognizing the constraints, it is essential to approach the findings with cautious optimism while emphasizing their unique contribution to a relatively uncharted domain.

**Table 4.** Normalized Percentages of Initiating Causes for EV Fires.

Major events for EV fire cause	Countries from where data of causes are taken from			Normalised average percentage of initiating causes of EV fire (%)	Number of fires per registered EV per year by causes (Normalised percentage of causes initiating the fire × 2.44E-04)
	Denmark %	Netherlands %	Sweden %		
S01: Human factors	16	11	7	9	1.90E-05
S02: Vehicles faults	49	25	32	29	5.77E-05
S03: Management faults	2	0	22	7	1.32E-05
S04: External factors	31	0	1	13	2.60E-05
S05: Unknown	2	64	37	42	8.41E-05
Total	100	100	100	100	2.44E-04

**Figure 4.**
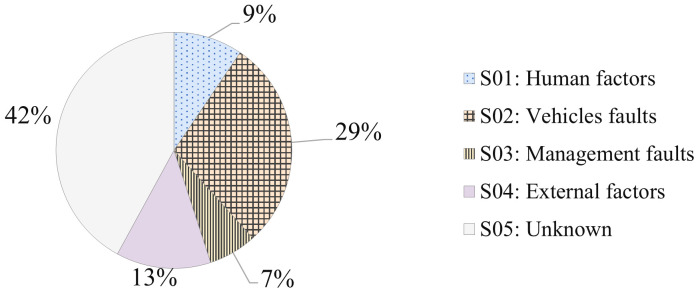
Proportional Representation of Initiating Causes for Electric Vehicle Fires.

Figure 4 demonstrates the proportional representation of initiating causes for EV fires based on Table 3. Vehicle Faults are discernible as a predominant cause, representing approximately 29% of incidents. This statistic emphasizes the necessity for rigorous regulatory oversight and enhanced quality assurance protocols in the realm of EVs. Human Factors, accounting for around 9%, exhibit noticeable disparities across countries. While these disparities might hint at cultural, educational, or infrastructural differences, drawing concrete conclusions is rendered intricate due to the aforementioned data acquisition challenges. Equally significant are the External Factors and Unknown Factors categories. The former, contributing to 13% of the dataset, possibly captures a spectrum of environmental and infrastructural elements. Meanwhile, the latter, with a considerable 42% contribution, accentuates the multifarious nature of EV fire incidents and the inherent challenges in pinpointing specific causes. Lastly, Management Faults, though comprising a smaller fraction at 7%, still present considerable variations across countries. This variance underscores the distinct management practices and post-incident procedural differences inherent to each nation. While the data provides a valuable preliminary insight into the causes of EV fires, the nuances and challenges tied to data acquisition across different countries warrant a measured interpretation of the findings.

Even though the normalized average percentage offers a somewhat unified perspective, the true value of this research lies in its pioneering nature. When juxtaposed with the number of fires per registered EV per year, the data, albeit limited, paints a crucial initial image of EV safety dynamics in these countries. In conclusion, while the data's scope is narrow, this research serves as a much-needed starting point in understanding the complex landscape of EV fires.

The primary limitation encountered in this analytical approach is the scarcity of data. Such a deficiency can potentially undermine the robustness of the conclusions drawn. While it is evident that sparse data may not provide a comprehensive representation of the real-world scenario pertaining to EV fires, it is imperative to underscore that this dataset, albeit limited, offers some foundational insights. In the absence of this data, the academic discourse would be devoid of any empirical evidence on the topic.

The constructed fault tree analysis illuminates the integral role of intermediate and basic causes. As such, these findings should serve as the cornerstone for data collection protocols implemented by first responders, particularly in the context of EV fires. This underscores the need for a homogeneous data acquisition approach, wherein uniform response mechanisms are employed across similar cohorts of responders. A case in point here would be the national fire and rescue services, who emerge as the ideal responders for such endeavours.

### Conclusions and recommendations

This study employs Fault Tree Analysis to systematically identify and assess the risks associated with EVs. Positioned at the juncture of innovation and vulnerability, four major causes for EV fires emerge: Human Factors, spanning from intentional actions like arson to accidents; Vehicle Factors, where battery defects and degradation take center stage; Management Factors, highlighting operational risks from charging to storage; and External Factors, such as wildlife interference or natural phenomena. To ensure a secure and sustainable EV era, a blend of technological advancements, rigorous safety protocols, public education, and adaptive strategies rooted in real-world data is imperative.

Furthermore, it becomes apparent that while various causes exist for EV fires, frequency offers a vital metric in truly assessing the scale of the challenge. More than just a statistic, this frequency encapsulates the probability of such incidents occurring and becomes an invaluable asset in quantitative fault tree analysis. In this study, a meticulous approach was adopted, employing a weighted average to calculate the annual EV fire frequency for each country. The resulting average annual EV fire frequency stood at 2.44 × 10 ^-4^ fires per registered EV, underscoring the importance of comprehensive data-driven insights. This frequency, underpinned by robust analytics, serves as a cornerstone in estimating potential EV fire risks based on registration numbers.

However, the inherent uncertainties in data quality and potential discrepancies in fire incident reporting emphasize the importance of ongoing research. Conclusively, while the road ahead in EV adoption is promising, this frequency serves as a pivotal benchmark, emphasizing the need for vigilance, adaptability, and continuous learning. In addition, understanding the frequency or probability of fire incidents using fault tree analysis provides the necessary tools for proactive risk management and strategic decision-making.

Building on the findings of this study, it is imperative to emphasize directions for future research. Firstly, as new battery technologies become integral to the transportation industry, it is essential to conduct in-depth studies to understand their inherent risks. Prompt research in this domain will catalyse the timely update of fire protection regulations in buildings. Secondly, the application of the Fault Tree Analysis extends beyond the confines of this study. It holds potential for two intricate scenarios that warrant thorough investigation: the fire protection measures for hydrogen fuel cell vehicles housed in buildings, and the safeguards surrounding second-generation electric batteries, which are already finding applications in architectural structures. These considerations are pivotal in shaping a comprehensive framework for fire safety in the evolving context of sustainable transportation and energy storage.

Finally, the importance of systematic and uniform data collection in the future cannot be overstated. Establishing the precise cause of an ignition source, particularly in the context of EV fires, often demands thorough investigative efforts. Unfortunately, not every incident is given this meticulous attention, leading to potential data discrepancies. To ensure that future studies and analyses benefit from the most accurate and relevant data, it's crucial to differentiate between fires "related to" and those "caused by" EVs. A fire simply being associated with an EV doesn't necessarily mean the vehicle instigated it. By taking a layered approach to data collection, starting with identifying the fundamental relationship of the fire to the EV and then delving deeper into its precise cause, we can ensure a more holistic view. Adopting this methodical approach will ensure a richer and more accurate data repository, fostering more informed decision-making and more effective preventive measures in the future.

**Figure 5.**
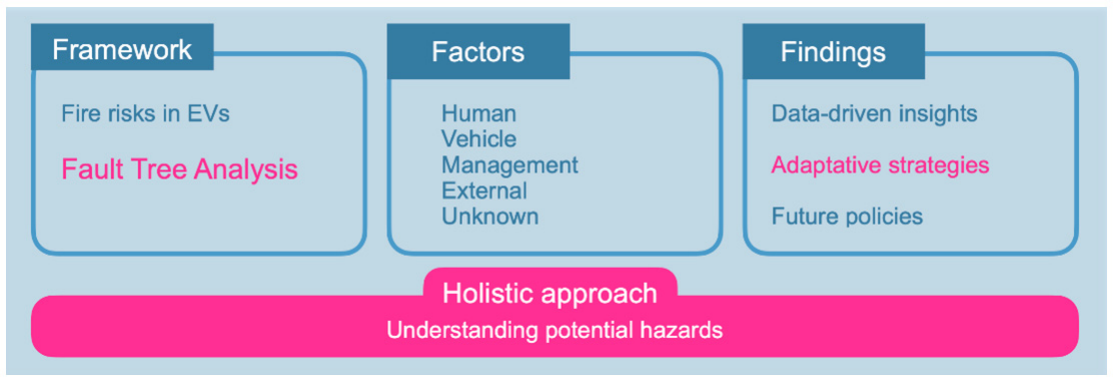
Graphical conclusions of the work.

In conclusion, Figure 5 graphically concludes that the work provided a comprehensive study on fire risks in EVs by employing a holistic approach and a Fault Tree Analysis to understand potential hazards. The analysis considered various factors, including human, vehicle, management, external, and unknown aspects, to derive data-driven insights. These insights are pivotal in developing adaptive strategies and shaping future policies to mitigate the identified risks and hazards.

## Data Availability

The data can be accessed from the following URL https://doi.org/10.5281/zenodo.8355177 Repository: Analysis of EV Fires from Accesible Public Domain Data https://doi.org/10.5281/zenodo.8355177 This project contains the following underlying data: •   Data file 1. EV Fire – Analysis.xlsx (The incidents of EV fires have been collated from publicly accessible data, aiming to offer insights into potential EV fires in the future. The dataset also includes causes as determined from the reports analyzed.) Repository: EV fire paper search string results https://doi.org/10.5281/zenodo.8398665 This project contains the following underlying data: •   Data file 1. EV Fire – Search Results.xlsx (EV fire paper search string results) Data are available under the terms of the Creative Commons Attribution 4.0 International license (CC-BY 4.0).
