# Supplementary material for: Electric vehicle fire risk assessment framework using Fault Tree Analysis
Source: Open Res Eur. 2023 Oct 18;3:178. [Version 1] doi: 10.12688/openreseurope.16538.1 (PMC10873543; doi:10.12688/openreseurope.16538.1)
Supplement: Peer review history

# Reviewer report: Reviewer response for version 1

**DOI:** 10.21956/openreseurope.17853.r37352

**Published:** 2024-02-22

**Authors:** Daniel Fruhwirt

**Affiliations:** 1 Graz University of Technology, Graz, Austria

The paper presents the assessment of electrical vehicle fires. Based on a literature study and data collection, a quantitative event tree analysis has been performed and basic and intermediate causes have been evaluated. The methodology for both literature and data study as well as the risk assessment are well explained. The authors highlighted advantages and weaknesses of the applied methodology in a proper way. The set of data only covers a manageable number of EV fires. This is not in the responsibility of the authors, as well prepared data are rare, but leads to uncertainties in statistics. In particular, the comparison of EV fire frequencies in different countries illustrates the sensibility of results depending on the available data. In traffic safety considerations, the applied methodology represents a common tool in order to identify causes and to enable measures that can be taken on basis of solid data. The results of the objective study are well presented and crucial aspects are properly explained.

 To summarize, the paper is well prepared, easy to read and deals with an important topic. The innovation of the presented study lies in the application of an established methodology on the topic of EV fires, which is already a hot topic and will attract more attention in the future. 

 However, I recommend some small changes of the current version:

1. Page 3, paragraph 4: "... to thermal runaway which could lead to whole vehicle to burn" 
  => to thermal runaway, which can cause the entire vehicle to burn.
2. Page 4, paragraph 5:  FCEVs are included in the definition of EVs, but for me it is not clear if the presented data also include hydrogen powered EVs.
  Personally, I prefer a separate treatment of BEVs and FCEVs as they represent different technologies. However, if you feel confident with the current definition please further explain or differentiate in table 3.
3. It is uncommon to use the number of fires per registered vehicles as an indicator of risk. In QRAs, usually the number of fires are related to the the mileage (or kilometres). 
4. Figure 4 has a poor quality. Please try to improve.
5. Page 10, paragraph 2: You mentioned that the presented numbers lean towards overestimation. I do not fully agree with this, because the presented data are quite new and aging processes on EVs will cause a higher number of EV fires compared to recent years. Please mention about the effect of aging that is not considered in the presented data.
6. Same paragraph: You mentioned that the number of EV fires will increase with an increase of registered vehicles. Your data in table 3 show the opposite. Norway and Sweden have the highest number of registered EVs, but the probability of an EV fire is the lowest (factor of 10). Please explain the possible cause.
7. Figure 3 presents the same information than table 3. Therefore, a deletion of Figure 3 could be considered, as it is not needed.

Is the study design appropriate and does the work have academic merit?

Yes

Is the work clearly and accurately presented and does it cite the current literature?

Yes

If applicable, is the statistical analysis and its interpretation appropriate?

Partly

Are all the source data underlying the results available to ensure full reproducibility?

Yes

Are the conclusions drawn adequately supported by the results?

Partly

Are sufficient details of methods and analysis provided to allow replication by others?

Yes

Reviewer Expertise:

My professional area covers all relevant aspects that are addressed in the paper.

I confirm that I have read this submission and believe that I have an appropriate level of expertise to confirm that it is of an acceptable scientific standard, however I have significant reservations, as outlined above.

# Reviewer report: Reviewer response for version 1

**DOI:** 10.21956/openreseurope.17853.r36197

**Published:** 2024-02-21

**Authors:** Ioanna A Koromila

**Affiliations:** 1 School of Naval Architecture and Marine Engineering, National Technical University of Athens, Athens, Greece

The paper employs fault tree analysis to estimate the fire risk of electric vehicles. It is important research that lays the foundation for the systematic investigation of electric vehicle fires using real accident data.

 A more representative title would be “Electric vehicle fire risk assessment using fault tree analysis”.

 In abstract-background, the abbreviation “EV” should be considered.

 In abstract-methods, it should be referred also to the performance of the quantitative FTA.

 In abstract-results, the expression "for each country" is not very clear because the description of the methodology where some country-level data are considered follows in the main text. A more accurate expression would probably be "per country". The same comment should apply throughout the manuscript reporting “EV fire frequency per country”.

 The keywords “state-of-art” and “architecture” are not relevant.

**In introduction:**

- Paragraph 1: A reference is required for “To achieve this goal,… from the year 2020”.
- Paragraph 2: Instead of “car transporter ship”, some other more relevant terms are “a ro-ro ship” or a “vehicle carrier”.
- Paragraph 2: Add “is assumed to be “ in the next expression “sank after an onboard fire, which is assumed to be started”.
- Paragraph 5: FTA is a “traditional tool” not a “critical tool”.
- Paragraph 5: Explanation of “PV” is needed.

**In methodology:**

- Paragraph 1: In the first expression it is better to write “risk assessment of EV fires”.
- Paragraph 3: The expression “In addition, …it instigated” shall be “In addition, the contribution of a specific fire cause was … ”.   
- Data acquisition: in line 6 is “selected”.
- Figure 1: the authors should provide the figure in higher resolution.
- Last paragraph: there is a repetition in reporting the period covering the data (first five lines need rewording).

**Results and discussion:**

- Paragraph 1: It is suggested to keep a similar form of referring numbers either “7 intermediate and 22 basic” or  ”seven intermediate and twenty two basic”. This is to be applied in the entire manuscript.
- Paragraph 2: “The first major event, human factors, is referred…”.
- Table 2: As understood, Event C12 does not correspond to “other”.
- Figure 2: In fault trees the direction of the vectors is "top-down" and not "bottom-up" as adopted by the authors.
- S02 Vehicle factors: In figure 2 it is referred as “vehicle faults”. The authors should change accordingly in the entire manuscript.
- It's noteworthy why the authors choose B3 and B4 not to be intermediate events since they seem to result from specific events. The authors should explain this.
- S03 Management factors: “The third major event, management factors, is related to …”.
- S03 Management factors: BESS, as it appears in figure 2, is not an intermediate event. The authors should also consider the relevant change in the graph (from rectangle to cyrcle). Moreover, the authors should also refer to C12 event.

**Quantitative fault tree analysis:**

- The last sentence of the first paragraph is not so clear.  
- The paragraph “further analysis….relatively uncharged domain” is not clear, rephrase is needed.
- Figure 4: the “management faults” shall be “management factors”.

Is the study design appropriate and does the work have academic merit?

Yes

Is the work clearly and accurately presented and does it cite the current literature?

Yes

If applicable, is the statistical analysis and its interpretation appropriate?

Not applicable

Are all the source data underlying the results available to ensure full reproducibility?

Yes

Are the conclusions drawn adequately supported by the results?

Yes

Are sufficient details of methods and analysis provided to allow replication by others?

Yes

Reviewer Expertise:

My expertise is in quantitative risk assessment with application on fire safety onboard ships.

I confirm that I have read this submission and believe that I have an appropriate level of expertise to confirm that it is of an acceptable scientific standard, however I have significant reservations, as outlined above.

# Reviewer report: Reviewer response for version 1

**DOI:** 10.21956/openreseurope.17853.r37370

**Published:** 2024-02-16

**Authors:** Guy Marlair

**Affiliations:** 1 French National Institute for Industrial Environment and Risks (INERIS), Verneuil-en-Halatte, France

This paper paper proposes an interesting approach making use of very classical fault tree analysis (FTA) tool -that is classically used in general risk analysis in the process industries. Such a tool is used here to provide a risk analysis framework as regard EV fires towards kind of  semi-quantitative evaluation of EV fire risk and key subsequent contributing factors and their categorization. The approach is based on a scientitic-sound literature review combining the use of peer reviewed papers and open sources. Authors are making use, rather cautiously, of accessible EV fire incident databases. Methods used, as well as incident data sources are well described and referenced, which is by far not always the case in many published papers relating to EV thermal runaway hazard and related fire hazard, in which scarce fire data are reported. 

 I just have some comments, here below listed for authors and readers of this paper.

 1. Clearly, EV fire statistics still need to be consolidated before achieving reliable statistics regarding EV battery failure rates: as partially explained by the authors, Incidents reports (in particular by the media), are rarely fully infomative and may convey misleading information. Sharp innovation in the battery sector, as well as cultural and legal aspects that differ significantly from country to country around the world are rendering comparisons quite difficult as well as the establishment of fully reliable statistics in the matter so far. As an illustration of that, when a majority of reports in the field seem to confirm lower EV annual fire frequencies as compared to ICE car fires, the contrary has sometimes been reported (see paper from Garreth Roberts entitled "Battery fire fears prompt thousands of recalls as EV interest increasing", in FleetNews Magazine, 19, 2020, page 15/78). 

 2. Statistical value of the reported annual frequency of EV fires in table 3 is relying on data from relatively small EV fleets (in relation with the size of the 6 countries where fire data could be accessed with enough confidence), therefore estimated fire frequency may be biased by missing fire data from large countries in which EVs have ben sold and used in significant quantities for some time such  China, USA, Canada... According to my knowledge, EV fire incidents in China are now counting in thousands annually , which figure has of course to be reported to the local cumulated registered EVs. 

 3. The idea of considering that a EV battery pack may be considered as a small "BESS" is only partially true in my opinion, since design constraints in particular are quite different, notably in terms of volumetric and massic energy densities of the battery system: this in turn may greatly influence the subsequent fire hazard. At system level, many other factors, even if the car is not on drive, can induce differences in the subsequent fire hazard (eg proportion of combustible components nearby the battery).

 4. I encourage the authors to look at the project led by Emma Sutcliffe (from Australia), see EV Fire safe web site and related infographics about EV fire statistics, that are regularly updated as far as fire statistics are consolidated with new data. In there, EV fire statistics are distributed with another choice of main contributing factors than in your table 4 and some discussion on the benchmarking could be useful for the readers.

 5. Although the analysis of the EV fire risk in this work is explicitly limited to passenger vehicle, it would be also useful to make a short comment about other vehicles/devices that are part of e-mobility: indeed, they are clearly much more of concern, as regard the fire risk, than passenger EV (see e-scooter and e-bike fires available statistics).

Is the study design appropriate and does the work have academic merit?

Yes

Is the work clearly and accurately presented and does it cite the current literature?

Yes

If applicable, is the statistical analysis and its interpretation appropriate?

Partly

Are all the source data underlying the results available to ensure full reproducibility?

Yes

Are the conclusions drawn adequately supported by the results?

Partly

Are sufficient details of methods and analysis provided to allow replication by others?

Yes

Reviewer Expertise:

general industrial safety, energy storage including battery safety issues, fire safety, fire testing, chemical/ material physical risk assessment

I confirm that I have read this submission and believe that I have an appropriate level of expertise to confirm that it is of an acceptable scientific standard, however I have significant reservations, as outlined above.

# Reviewer report: Reviewer response for version 1

**DOI:** 10.21956/openreseurope.17853.r37353

**Published:** 2024-02-16

**Authors:** J. Yoon Choi

**Affiliations:** 1 Korea Conformity Laboratories, Seoul, South Korea

Applying fault tree analysis, the causes of electric vehicle fire risks were discerned. Five major risk factors and related issues were suitably analysed.

 Applying public domain data, the five deduced causes of electric vehicle fire causes were analysed and the conclusion was reasonably drawn. 

 However, based on the public accidental information, if the related issues of each risk factors were analysed, this article could have better impact to the related researchers.

Is the study design appropriate and does the work have academic merit?

Yes

Is the work clearly and accurately presented and does it cite the current literature?

Yes

If applicable, is the statistical analysis and its interpretation appropriate?

Yes

Are all the source data underlying the results available to ensure full reproducibility?

Yes

Are the conclusions drawn adequately supported by the results?

Yes

Are sufficient details of methods and analysis provided to allow replication by others?

Yes

Reviewer Expertise:

battery fire, Electric vehicle fire related fire sciences

I confirm that I have read this submission and believe that I have an appropriate level of expertise to confirm that it is of an acceptable scientific standard.

# Reviewer report: Reviewer response for version 1

**DOI:** 10.21956/openreseurope.17853.r36200

**Published:** 2024-02-16

**Authors:** Jun Liu

**Affiliations:** 1 The University of Alabama, Tuscaloosa, Alabama, USA

This study provides a framework for EV fire risk assessment. I think this topic is very important and timely, and stakeholders/communities need to prepare for the arrival of massive EVs. My largest concerns about this study include:

 1. This study is based on the historical EV incidents. It is very valuable to look back and see what has happened. However. how would the information remain still valuable for next 5 or 10 years. I understand that this study could be limited by the data availability. It would be great if authors could show a temporal trend of these factors that lead to EV fires?

 2. EVs, to me, are an emerging technology and there are may rapidly evolve in next decade. Think about the battery technologies, and vehicle automation/connectivity technologies. All these factors may need to be explicitly discussed in this paper, and show how these factors would affect the results i.e. the EV fire rate.

 3. The fault tree analysis is the most concerned part. It is unclear to me that how the factors are grouped together. Human factors and crashes. It might be good to group EV fires based on their locations (basically, whether on roads, or not on roads). What matters a lot to me is the EV fires on roads which could lead to other consequences about traffic incident management and secondary crashes. I realize that authors aim to include all EV fires. I would appreciate authors could go deeper to different types of EV fires.

Is the study design appropriate and does the work have academic merit?

Partly

Is the work clearly and accurately presented and does it cite the current literature?

Yes

If applicable, is the statistical analysis and its interpretation appropriate?

Not applicable

Are all the source data underlying the results available to ensure full reproducibility?

Yes

Are the conclusions drawn adequately supported by the results?

Partly

Are sufficient details of methods and analysis provided to allow replication by others?

Partly

Reviewer Expertise:

transportation engineering

I confirm that I have read this submission and believe that I have an appropriate level of expertise to confirm that it is of an acceptable scientific standard, however I have significant reservations, as outlined above.

# Reviewer report: Reviewer response for version 1

**DOI:** 10.21956/openreseurope.17853.r35687

**Published:** 2023-11-14

**Authors:** Amaya Osacar

**Affiliations:** 1 Knauff Installation, Visé, Belgium

The promotion of electric vehicles (EV) is among the global efforts to reduce Green House Gases (GHG) emissions. The growing number of EVs has brought about concerns regarding the EV related fire hazard.  

 The article proposes a suitable method to address the specific characteristics of the fire risk associated to this type of vehicle. The methodology is applied to available public domain information.  

 The most relevant causes and its frequency are identified through qualitative Fault Tree Analysis of the data. Calculation of the average annual EV fire rate, based on weighted average number of fires per year, estimates 2.44 × 10-4 fires per registered EV. 

 The work provides a comprehensive study on fire risks in EVs. The proposed methodology allows the assessment of both the probability and inherent risk of such incidents.

Is the study design appropriate and does the work have academic merit?

Yes

Is the work clearly and accurately presented and does it cite the current literature?

Yes

If applicable, is the statistical analysis and its interpretation appropriate?

Yes

Are all the source data underlying the results available to ensure full reproducibility?

Yes

Are the conclusions drawn adequately supported by the results?

Yes

Are sufficient details of methods and analysis provided to allow replication by others?

Yes

Reviewer Expertise:

Fire safety (buildings, products, policies, standards, regulatory frameworks).

I confirm that I have read this submission and believe that I have an appropriate level of expertise to confirm that it is of an acceptable scientific standard.
